# Dietary tetrahydrocurcumin reduces renal fibrosis and cardiac hypertrophy in 5/6 nephrectomized rats

**DOI:** 10.1002/prp2.385

**Published:** 2018-02-19

**Authors:** Wei Ling Lau, Mahyar Khazaeli, Javad Savoj, Kasim Manekia, Maria Bangash, Roshni G. Thakurta, Anhthu Dang, Nosratola D. Vaziri, Bhupinder Singh

**Affiliations:** ^1^ Division of Nephrology and Hypertension Department of Medicine University of California‐Irvine Orange CA; ^2^ Department of Internal Medicine Riverside Community Hospital University of California‐Riverside School of Medicine Riverside CA; ^3^ New York Medical College Valhalla NY

**Keywords:** chronic kidney disease, fibrosis, hypertension, tetrahydrocurcumin

## Abstract

Tetrahydrocurcumin (THC) is the principal metabolite of curcumin and has antioxidant properties. In the present investigation, the effect of THC on renal and cardiovascular outcomes was studied in rats with chronic kidney disease (CKD). CKD rats were randomized following 5/6 nephrectomy to a special diet for 9 weeks which contained 1% THC (CKD+THC group). Low‐dose polyenylphosphatidylcholine was used as a lipid carrier to increase bioavailability. Endpoints included tail blood pressure, normalized heart weight, plasma and urine biochemical data, and kidney tissue analyses. CKD animals demonstrated increased proteinuria, decreased creatinine clearance, hypertension, and cardiac hypertrophy. The antioxidant proteins CuZn SOD and glutathione peroxidase were decreased in the remnant kidney, while apoptosis (caspase‐3) and fibrosis (alpha‐SM actin) were increased. Renal fibrosis was confirmed histologically on trichrome staining. These pathologic changes were ameliorated in the CKD+THC group with significant decrease in proteinuria, hypertension, and kidney fibrosis. THC therapy restored levels of CuZn SOD and glutathione peroxidase. Consistent with prior reports, dietary THC did not improve nuclear Nrf2 levels. In summary, dietary THC therapy improved expression of antioxidant proteins in the remnant kidney, decreased renal fibrosis and proteinuria, and ameliorated hypertension in 5/6 nephrectomized rats.

**Abbreviations**
BP: blood pressure
BUN: blood urea nitrogen
CKD: chronic kidney disease
CrCl: creatinine clearance
CRP: C‐reactive protein
PPC: polyenylphosphatidylcholine
ROS: reactive oxygen species
THC: tetrahydrocurcumin

## 1 INTRODUCTION

The pathogenesis of chronic kidney disease (CKD) involves a complex interaction of inflammation, oxidative stress, and fibrosis that lead to progressive glomerular and tubulointerstitial scarring.^1^, ^2^ Oxidative stress promotes inflammation via toxic effects of reactive oxygen species (ROS) and by activation of redox‐sensitive proinflammatory signaling pathways. Besides driving kidney failure, inflammation and oxidative stress perpetuate atherosclerosis and vascular calcification, thus directly contributing to the elevated cardiovascular morbidity and mortality rates in CKD patients.^2^, ^3^

Curcumin (diferuloylmethane; 1,7‐bis[4‐hydroxy‐3‐methoxyphenyl]‐1,6‐heptadiene‐3,5‐dione) is the major bioactive component of the herb turmeric or *Curcuma longa L*., a widely used natural food product in curry powder and food coloring (mustard). The principal metabolite of curcumin is tetrahydrocurcumin (THC), produced by medium‐chain dehydrogenase/reductase metabolism of enteric bacteria.^4^ Curcumin has been studied in a variety of settings including cancer, inflammatory bowel disease, neurogenesis, and diabetes mellitus.^5^, ^6^, ^7^, ^8^, ^9^ While it has strong anti‐inflammatory and antioxidant properties, curcumin has been shown to exert some prooxidant activity when tested in tumor cell lines.^10^ THC has been reported to exhibit similar antioxidant properties 11 without the unfavorable prooxidant effects, and may be superior in the induction of glutathione peroxidase and quenching of free radicals.^12^

Beneficial antioxidative effects of THC have been reported in acute kidney injury models. THC prevented ferric nitrilotriacetate and chloroquine‐induced oxidative kidney injury via inhibition of lipid peroxidation and upregulation of antioxidant catalytic activity.^12^, ^13^ In rats with streptozotocin‐induced diabetes mellitus, THC has been shown to decrease tissue oxidative stress, decrease albuminuria and serum creatinine, and improve plasma insulin levels.^14^, ^15^, ^16^ Curcumin but not THC has previously been investigated in 5/6 nephrectomized CKD rats 17, 18, 19 and we chose to study THC in this nephron mass reduction model to determine effects on proteinuria, fibrosis, and inflammation.

THC is more stable than curcumin, with a degradation half‐life in cell culture medium of 813 minutes as compared to 186 minutes for curcumin.^20^ While THC has better enteric absorption than curcumin, both molecules are poorly water‐soluble; thus, bioavailability is a major issue.^21^ Delivery of curcumin with a lipid carrier such as polyenylphosphatidylcholine (PPC) has been shown to increase plasma levels fivefold in rats.^22^

Our current project investigated effects of dietary 1% tetrahydrocurcumin in the well‐established rat 5/6 nephrectomy model. A low concentration of PPC was added to enhance curcuminoids bioavailability. We report that THC with PPC had positive renal effects, which correlated with decreased systemic hypertension and less cardiac hypertrophy.

## 2 MATERIALS AND METHODS

### 2.1 Experimental animals

**Figure 1:**
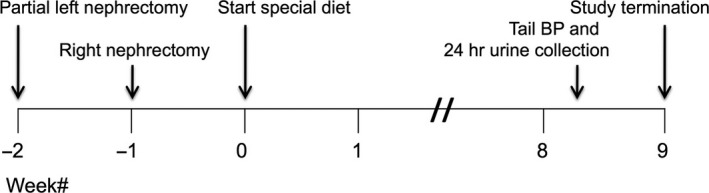
Experimental timeline. Study groups examining dietary tetrahydrocurcumin (THC) therapy in CKD rats

Female Sprague‐Dawley rats (body weight 225‐250 g, Charles River, Wilmington, MA) were randomized to CKD and control groups. The experimental groups were: control (CTL, n = 6), CKD (n = 9), and CKD treated with THC diet (CKD+THC, n = 10). Female rats were chosen as they develop more proteinuria and renal fibrosis when subjected to 5/6 nephrectomy, as compared to male rats.^23^ The CKD group underwent 5/6 nephrectomy: resection of the 2 poles of the left kidney followed by right total nephrectomy 1 week later. Surgeries were done under inhaled isoflurane anesthesia and rats were given buprenorphine 0.05 mg/kg i.p. at the start of surgery for analgesia. CKD rats were randomized 10 days after right nephrectomy to regular chow or special diet × 9 weeks. Tail blood pressure (BP) measurements and 24 hours urine collections (within individual metabolic cages, 6 animals per group) were done within 1 week prior to study termination. All experimental protocols were approved by the University of California, Irvine Institutional Committee for the Use and Care of Experimental Animals. The experimental timeline is shown in Figure 1.

### 2.2 Tetrahydrocurcumin Diet

We chose a 1% THC diet based on review of the literature that showed measurable physiologic effects with 0.5%‐4% dietary curcumin.^24^, ^25^, ^26^, ^27^, ^28^ Diets were based on the 2020X regular rodent chow (Teklad Diets, Madison WI). Additional diet components were tetrahydrocurcumin (curcumin C3 reduct with tetrahydrocurcuminoids 95%, Sabinsa Corporation, Payson UT) and polyenylphosphatidylcholine (PPC, PhosChol Liquid Concentrate whereby 1 teaspoon contains 3000 mg of purified PPC, Nutrasal, Inc., Scottsdale AZ). The 1% THC diet was manufactured into pellet form by Teklad Diets: diet formulation TD.140853 contained 1% tetrahydrocurcumin + PPC 3 g/1000 kcal or 9.3 g/kg of diet. PPC added to the diet served as a lipid carrier to increase bioavailability of the curcumin compounds^22^ and the dose 3 g/1000 kcal is well‐tolerated in rats.^29^, ^30^ This dose is approximately 100‐fold lower than doses reported to have renoprotective effects.^31^, ^32^

### 2.3 Tissue harvest

Rats were euthanized after 9 weeks on diet by exsanguination using cardiac puncture under general anesthesia. Kidney and heart tissues were harvested and processed for western blot analysis and histology. Plasma was aliquoted for biochemical analysis and mass spectrophotometry assays.

### 2.4 Blood and urine biochemical data

Hemoglobin was measured using an automated meter (Germaine Labs AimStrip Hemoglobin Test System, catalog# 23‐111‐280, Fisher Scientific). Plasma samples were analyzed for blood urea nitrogen (BUN) and creatinine using colorimetric kits from BioAssay Systems (Hayward CA). Urine samples were diluted 30x for creatinine (BioAssay Systems) and 5x for protein measurements (Rat Urinary Protein Assay Kit, Chondrex, Inc., Redmond WA). Creatinine clearance normalized to body weight (CrCl, mL/min*kg) was calculated from 24 hours urine collections using the formula: [urine Cr x urine volume]/[serum Cr x 1440 x body weight]. Plasma C‐reactive protein (CRP) and galectin‐3 were measured using rat ELISA kits from Sigma‐Aldrich (St. Louis MO) and MyBioSource (San Diego CA).

### 2.5 Mass spectrometry for tetrahydrocurcumin

Analysis was done at the Mass Spectrometry facility at University of California, Irvine's Chemistry department. We used a modified protocol based on previously described methods.^33^, ^34^ A 0.1 mL aliquot of plasma was treated with 100 μL of a solution containing 1000 U of beta‐glucuronidase/sulfatase (EC 3.2.1.31) from *Helix pomatia* (catalog# G7017‐1ML, Sigma‐Aldrich) in 0.1 mol/L phosphate buffer. The mixture was vortexed and incubated at 37°C for 1 hour to hydrolyze the phase‐2 conjugates of curcuminoids. After incubation, curcuminoids were extracted with 1 mL of ethyl acetate and the mixture was vortexed for 1 minute, followed by sonication in a water bath for 15 minutes. The mixture was subjected to centrifugation at 15,000 g for 6 minutes and the upper organic layer was transferred to a 2 mL microcentrifuge tube and evaporated to dryness at 30°C in a Speed Vac. This extraction process was repeated, for a total of 2 extractions, yielding 1 dried extract which was reconstituted in 100 μL of 50% acetonitrile solution with 0.1% formic acid. The tube was sonicated in a water bath for 30 minutes to ensure dissolution.

Tetrahydrocurcumin reference standard fluka 50202‐10MG was purchased from Sigma‐Aldrich. A stock 20 mg/mL solution was prepared with acetonitrile and dilutions were prepared with deionized water to generate a standard curve ranging 0.25‐1000 μg/mL. Standards and prepared samples were injected (10 μL) into the HPLC‐MS/MS instrument, Waters Quattro Premier XE equipped with UPLC. The UPLC has a BEH C18 column which allows rapid sample throughput. Mobile phase A was 2% acetonitrile with 0.2% acetic acid, and mobile phase B was 100% acetonitrile with 0.2% acetic acid. Analysis was performed using multiple reaction monitoring MS/MS with standard calibration. The tetrahydrocurcumin transition was *m/z* 373.0 → 136.8.

### 2.6 Western blot analysis

Tissue lysates were prepared from kidney samples using Tissue Extraction Reagent I (Thermo Fisher Scientific, Waltham MA) supplemented with cOmplete Protease Inhibitor Cocktail (Roche Diagnostics Corp., Indianapolis IN). Protein concentration in the tissue homogenates was determined by bovine serum albumin assay kit (Pierce, Rockford IL) and 50 μg of total protein per sample was fractionated on 4%‐12% Bis‐Tris gradient gel (Invitrogen, Carlsbad CA) then transferred to a polyvinylidene fluoride membrane. The membrane was blocked with 5% nonfat dry milk solution in Tris‐buffered saline with 0.05% Tween, then incubated with the following primary antibodies: Cu/Zn SOD (final concentration 33.5 μg/mL, catalog# 574597 from Calbiochem/EMD Millipore, Billerica MA), alpha‐smooth muscle actin (1:200 dilution, A5228 from Sigma‐Aldrich), iNOS (16 μg/mL, PA1‐036 from Thermo Scientific), glutathione peroxidase‐1 (1:200 dilution, AF3798 from R&D Systems, Minneapolis, MN), COX‐2 (1:200 dilution, ab15191 from Abcam), caspase‐3 (1:250 dilution, ab90437 from Abcam), catalase (4 μg/mL, C0979 from Sigma‐Aldrich). Beta‐actin antibody (Sigma‐Aldrich) at 1:10 000 or GAPDH (Abcam) at 1:20 000 was used to standardize the data. Nuclear lysates were prepared using the NE‐PER kit from Thermo Scientific and probed for Nrf2 (1 μg/mL, SAB4501984, Sigma) with histone H3 (1:5000 dilution, ab1791, Abcam) for standardization. The appropriate horseradish peroxidase‐conjugated secondary antibodies (Sigma‐Aldrich) were used at 1:20 000 dilution. The membrane was visualized with SuperSignal West Pico (Pierce) and developed by autoluminography. Band densities were quantified using ImageJ software (version 10.2) from the National Institutes of Health (www.imagej.nih.gov/ij/).

### 2.7 Histopathological analysis

Paraffin sections of kidney and heart tissue were deparaffinized with xylene, dehydrated in alcohol series, stained with hematoxylin and eosin (H&E) or Masson's trichrome, and examined under a photomicroscope (Nikon Eclipse, Japan). An ImageJ macro was used for quantification of kidney fibrosis (% area stained blue on Masson's trichrome) while maintaining the same threshold settings across all slides to avoid bias.^35^ Three images of kidney cortex were captured at 10X objective from 6 animals per group by a researcher blinded to the study groups (RGT), and mean % area was calculated per animal.

### 2.8 Statistical analysis

Data were screened for outliers using the Grubbs’ test (extreme studentized deviate method, http://graphpad.com/quickcalcs/grubbs1/). Bartlett's test was used to assess homogeneity of variances across groups. For datasets with equal variances, group data were analyzed using one‐way ANOVA with post hoc Tukey, and *P* < .05 was considered significant. For nonparametric data, Kruskal‐Wallis analysis was used (*P* < .05 considered significant) with Dunn's Multiple Comparison Test. Data are reported as mean ± SD for plasma and urine biochemical data and weights, and mean ± SEM for western blot, histologic and mass spectrometry quantitation. Figures were generated using GraphPad Prism 4 software (GraphPad Software, San Diego CA).

## 3 RESULTS

### 3.1 General Data

**Figure 2:**
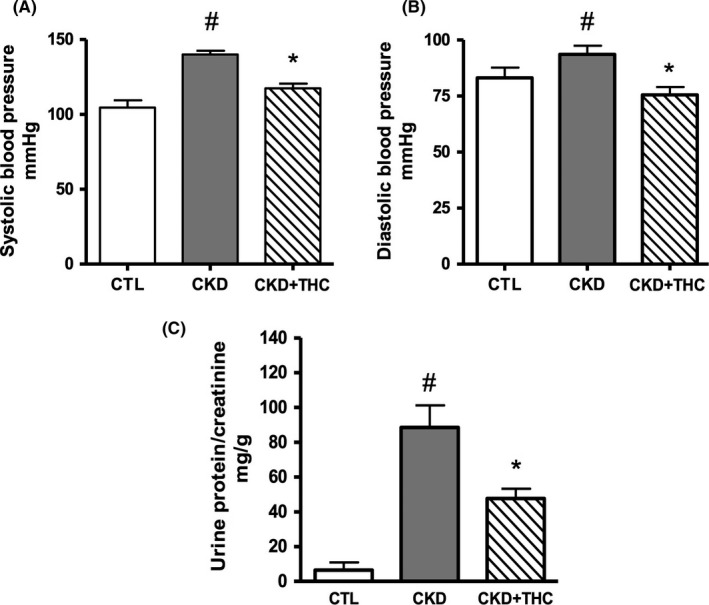
Blood pressure and proteinuria were decreased with tetrahydrocurcumin (THC) therapy. Systolic blood pressure (A) and diastolic blood pressure (B) were increased in CKD rats, and decreased with THC therapy. Proteinuria as assessed by urine protein/creatinine ratio (C) was significantly elevated in 5/6 nephrectomized CKD animals, and was significantly decreased with THC therapy. Data shown as mean ± SEM. ^#^ *P* < .05 vs CTL; *P < .05 vs CKD

At study conclusion, there were 6 rats in the normal control group (CTL), 6 rats in the nontreated CKD group, and 9 rats in the 1% tetrahydrocurcumin/PPC group (CKD+THC). There were 3 deaths in the nontreated CKD group and 1 death in the CKD+THC group during the 9 weeks on assigned diet. Our study was not powered to detect mortality differences. Table 1 and Figure 2 summarize plasma and urine biochemical data, body weights, heart weights, and tail blood pressure values. BUN at week 0 and week 9 of assigned diet and plasma creatinine were significantly elevated in the CKD groups compared to CTL, while creatinine clearance (CrCl) was significantly decreased. Body weight, hemoglobin, plasma CRP, and galectin‐3 were not significantly different between groups, although average CRP was highest in the nontreated CKD group. There was a trend for improved BUN, creatinine, and CrCl in the THC treatment groups, although this did not reach statistical significance.

**Table 1:** Body weights, normalized heart weights, and blood and urine biochemical data for the 4 experimental groups. Data are shown as mean ± SD

	CTL n = 6	CKD n = 6	CKD+THC n = 9
Body weight (g)	282 ± 18	260 ± 13	257 ± 11
Heart weight/body weight (g/kg) a	3.5 ± 0.3	4.6 ± 0.5^b^	4.3 ± 0.8
Hemoglobin (g/dL)	14.1 ± 0.5	13.7 ± 1.1	12.3 ± 1.5
BUN at start of special diet (mg/dL)	N/A	63 ± 10	69 ± 15
Terminal BUN (mg/dL) a	20.6 ± 3.4	87.1 ± 32.9^b^	61.3 ± 25.5^b^
Plasma creatinine (mg/dL) a	0.4 ± 0.1	1.4 ± 0.5^b^	1.1 ± 0.2^b^
CrCl (mL/min*kg)	5.2 ± 1.2	1.8 ± 1.1^b^	2.8 ± 0.7^b^
24 h total urine protein (mg) a	18 ± 17	663 ± 252^b^	528 ± 119
Plasma C‐reactive protein (mg/mL)	1.9 ± 1.1	2.8 ± 1.2	2.7 ± 0.9
Plasma galectin‐3 (ng/mL)	444 ± 148	475 ± 162	400 ± 208

BUN*,* blood urea nitrogen; CrCl, creatinine clearance; CKD, chronic kidney disease; CTL, controls; THC, tetrahydrocurcumin/PPC diet.

a Data with unequal variances by Bartlett's test, analyzed using Kruskal‐Wallis test.

b *P* < .05 vs CTL.

Tail systolic and diastolic BP readings were significantly elevated in 5/6 nephrectomized rats and this hypertension was ameliorated in CKD rats on THC diet (Figure 2). The CKD+THC rats also had a significant decrease in proteinuria by ~50% compared to CKD animals (47.6 ± 13.8 vs 88.5 ± 30.9 mg protein per g creatinine). Furthermore, cardiac hypertrophy (assessed by heart weight normalized to body weight) was decreased by THC therapy such that normalized heart weight was not significantly different in this group vs CTL animals.

### 3.2 Mass Spectrometry Analysis

THC was not detected in the plasma from CTL and nontreated CKD rats. Average plasma THC level was 24.0 ± 3.3 μg/mL (0.06 mmol/L) in CKD+THC rats.

### 3.3 Kidney western blot data

**Figure 3:**
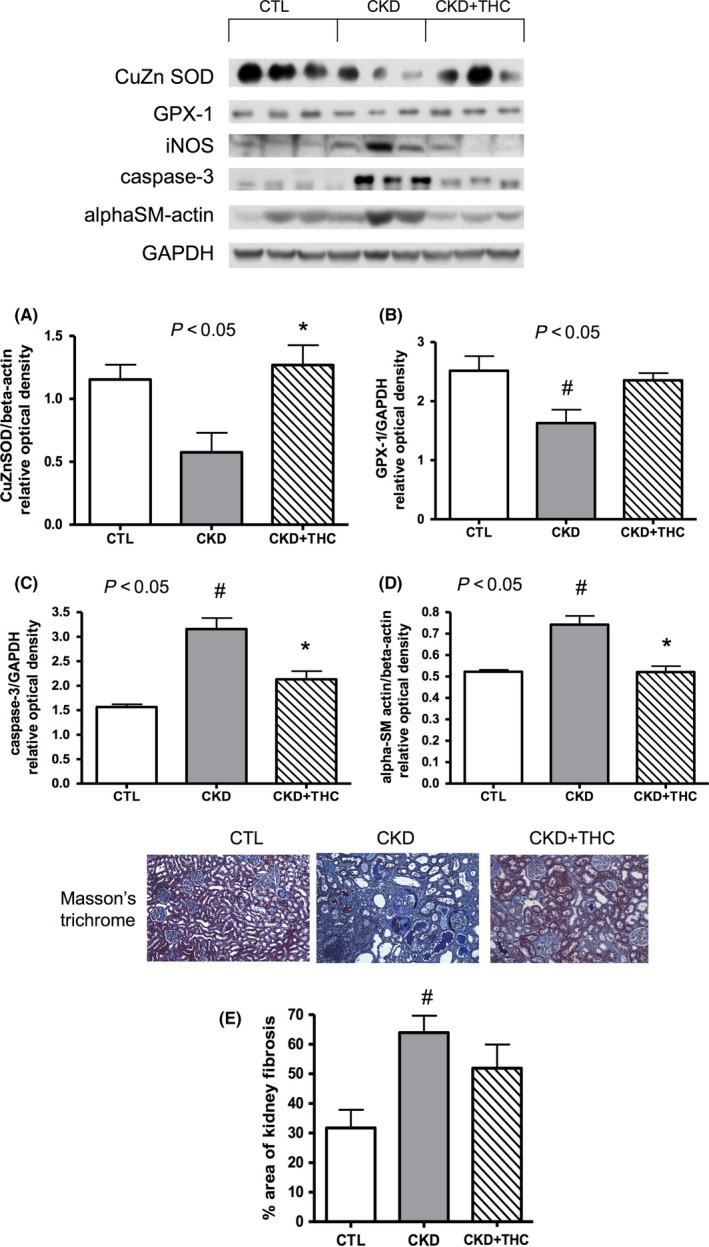
Improvement in markers of oxidative stress on western blot and histology. Western blots depicting protein abundance of oxidative stress, inflammation, and fibrosis mediators in kidney lysates from the 3 experimental groups. The antioxidant (scavenging) proteins (A) copper‐zinc superoxide dismutase (CuZn SOD), and (B) glutathione peroxidase (GPX‐1) were decreased in CKD and levels were restored with dietary THC therapy. The apoptosis marker (C) caspase‐3 and the fibrosis marker (D) alpha‐smooth muscle actin (alphaSM‐actin) were increased in the remnant kidney from CKD rats and were decreased with THC therapy. (E) Representative micrographs of kidney tissue from the 3 experimental groups, stained with Masson's trichrome to assess degree of fibrosis (percent area stained blue, 10X objective). Area stained blue was doubled in CKD vs CTL animals. This area was decreased ~20% with THC therapy. Data shown as mean ± SEM. ^#^ *P* < .05 vs CTL; *P < .05 vs CKD

Western blot analysis of markers of oxidative stress, apoptosis, and fibrosis in kidney lysates is summarized in Figure 3. The antioxidant (scavenging) enzymes metalloenzyme copper‐zinc superoxide dismutase (CuZn SOD) and glutathione peroxidase (GPX‐1) were suppressed in the remnant kidney from CKD animals. Levels were restored with dietary THC treatment. Another cytoplasmic antioxidant enzyme, catalase, was similarly suppressed in CKD and there was a trend for improved levels with THC diet (*P* = .24 across groups, data not shown). Inducible nitric oxide synthase (iNOS) was increased in CKD and there was a trend for decreased levels with THC diet (*P* = .09); COX‐2 levels were not different across CKD groups (data not shown). Consistent with prior work that has shown THC to be a weak inducer of Nrf2 translocation compared to its parent compound curcumin,^36^ nuclear Nrf2 levels were not increased with THC treatment (data not shown). Apoptosis and fibrosis were increased in the remnant kidney from CKD rats as indicated by elevated caspase‐3 and alpha‐smooth muscle actin (alphaSM‐actin), and these markers were decreased with THC diet.

### 3.4 Histology results

Masson's trichrome was used to visually assess percent area affected by fibrosis (stained blue) in kidney tissues. Three cortical micrographs per kidney section were assessed using an ImageJ macro and an average was calculated per animal; 6 animals were assessed per experimental group. Percent area stained blue was doubled in the CKD animals compared to CTL rats (63.9% vs 31.7%) and was decreased ~20% with THC treatment (51.9%). Representative kidney sections and group data are shown in Figure 3E. No increase in fibrosis was observed in the heart tissue from the CKD groups, compared to CTL rats (data not shown).

## 4 DISCUSSION

**Figure 4:**
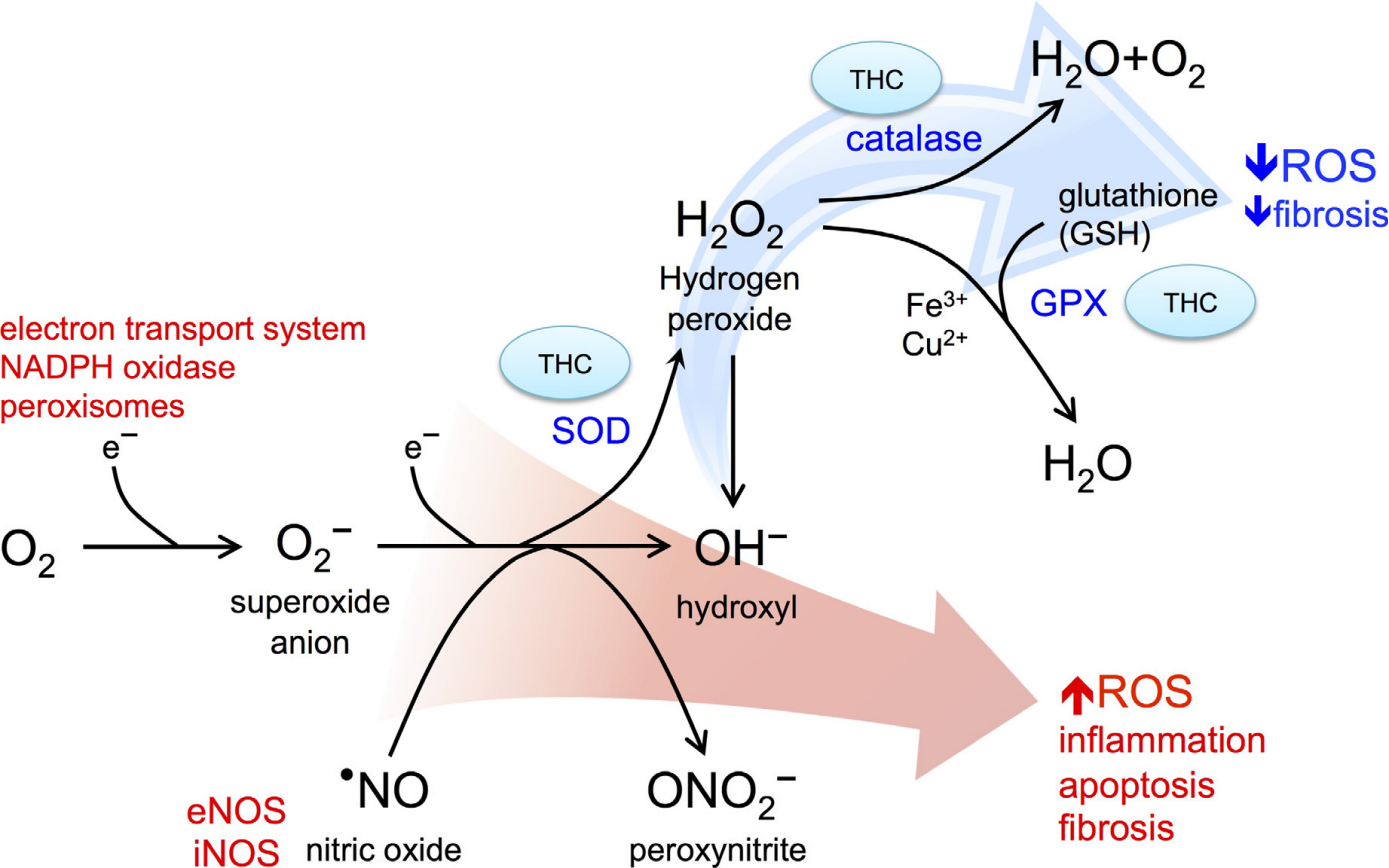
Summary of pathways by which tetrahydrocurcumin (THC) decreases oxidative stress and renal fibrosis. There is heightened oxidative stress in CKD and transfer of electrons by mitochondrial, peroxisomes, nicotinamide adenine dinucleotide phosphate (NADPH), and nitric oxide synthase (NOS, inducible iNOS and endothelial eNOS) reduction pathways results in generation of damaging reactive oxygen species (ROS) including superoxide, nitric oxide, peroxynitrite, and hydroxyl ions. This oxidative stress promotes inflammation, cell death, and fibrosis. THC restores expression of antioxidant (scavenging) proteins including superoxide dismutase (SOD), catalase, and glutathione peroxidase (GPX) to decrease levels of ROS, thus decreasing downstream fibrosis

To the best of our knowledge, this is the first study of dietary tetrahydrocurcumin in 5/6 nephrectomy rats. THC treatment had beneficial renal and cardiac effects. CKD animals exhibited hypertension, proteinuria, renal fibrosis, and cardiac hypertrophy; dietary THC improved all of these parameters. Chronic oxidative stress and inflammation are hallmarks of CKD^37^, ^38^; in this study, levels of antioxidant enzymes CuZn SOD and GPX were decreased, while inflammatory iNOS and the apoptosis mediator caspase‐3 were increased in the remnant kidney tissues from CKD rats. These derangements were corrected with 1% THC diet. The proposed pathways by which THC improves oxidative stress and decreases apoptosis and fibrosis are summarized in Figure 4.

CKD is a state of SOD deficiency whereby the latter promotes blood pressure dysregulation and nitric oxide metabolism.^39^ The observed renoprotective effects in our study are consistent with reports from other rodent models whereby THC enhanced antioxidative activity of superoxide dismutase, catalase and glutathione peroxidase.^12^, ^13^, ^16^ The net effect is decreased production of reactive oxygen species, with subsequent downregulation of the caspase‐3 apoptosis pathway^40^ and tissue fibrosis. THC has similarly been shown to decrease albuminuria and blunt rise in serum creatinine in rats with diabetic nephropathy.^14^, ^15^, ^16^

The cardioprotective outcomes (normalized blood pressure, decreased cardiac hypertrophy) were likely secondary to decreased severity of CKD, but may have also reflected direct myocardial benefits of THC in terms of suppressing ROS production, apoptosis, and fibrosis. Curcumin therapy has been associated with decreased cardiac oxidative stress and improved ejection fraction in models of CKD, obesity, and cardiac ischemia/reperfusion.^41^, ^42^, ^43^ We further examined circulating galectin‐3 levels given the strong evidence that this beta‐galactoside‐binding lectin promotes tissue fibrosis.^44^, ^45^ We found no differences in galectin‐3 levels between the experimental rat groups, suggesting that galectin‐3 was not a significant instigator of renal fibrosis and is not a useful biomarker in this CKD model.

The decrease in proteinuria with THC therapy is noteworthy as increased proteinuria promotes fibrosis and more rapid loss of kidney function in CKD patients^46^, ^47^ and associates with higher cardiovascular mortality.^48^ Anti‐proteinuric interventions are lacking in clinical practice, other than pharmacologic blockade of the renin‐angiotensin‐aldosterone system.^49^, ^50^ Concurrent with decreased proteinuria, we noted a trend for improved kidney function as evidenced by lower BUN and plasma creatinine, and higher creatinine clearance in the THC‐treated animals. We postulate that this effect may have reached statistical significance if the THC diet had been started immediately after the second surgery (Figure 1). Other groups have reported that THC significantly decreased serum creatinine in nonsurgical kidney injury models, for example, when given at the time of chloroquine‐induced oxidative stress.^12^, ^13^

The use of PPC as a lipid carrier increased intestinal uptake of curcuminoids and yielded robust THC plasma levels, averaging 0.06 mmol/L in the CKD+THC treatment group. Aside from boosting enteric absorption of curcuminoids, PPC has been reported to have beneficial cytoprotective effects in the liver and kidney.^30^, ^31^, ^32^ Of note, prior studies used much higher doses of oral PPC (100 mg/kg body weight),^31^, ^32^ whereas our present study used a concentration of 9.3 g/kg of diet which has been shown to have protective effects against alcohol‐induced liver oxidative injury.^29^, ^30^ Assuming that a 250 g adult rat eats ~25 g of chow per day, this approximates a PPC dose of 0.2 g/day or 0.93 mg/kg body weight ,that is, 100‐fold lower than the reported renoprotective dose.^31^, ^32^

Future studies will need to examine effects of altered gut permeability on THC bioavailability. Altered intestinal microbiome in CKD induces gut inflammation and degradation of epithelial tight junctions leading to a “leaky” barrier, with subsequent translocation of luminal toxins into the bloodstream.^51^ In prior CKD rat studies, uremia was associated with increased gut permeability to polyethylene glycols ranging up to 1162 Da in size.^52^ THC has a molecular weight of 372.4 Da and thus theoretically could be subject to increased gut translocation in CKD. However, curcuminoids have been reported to decrease intestinal barrier dysfunction in cell culture models^53^ and this could negate any increase in THC uptake. Further in vivo studies are needed to clarify the net drug bioavailability.

In summary, 1% tetrahydrocurcumin with PPC diet improved expression of antioxidant enzymes in the remnant kidney, decreased renal apoptosis and fibrosis, and ameliorated proteinuria, hypertension, and cardiac hypertrophy in 5/6 nephrectomized rats. Further studies are needed to investigate whether these beneficial effects of THC therapy can be replicated in patients with CKD.

## ACKNOWLEDGEMENTS

The study was funded by an unrestricted research grant from Hub Therapeutics LLC, Phoenix, AZ.

## DISCLOSURE

None declared.
